# In vitro and in vivo pharmacological activity of minor cannabinoids isolated from *Cannabis sativa*

**DOI:** 10.1038/s41598-020-77175-y

**Published:** 2020-11-23

**Authors:** Ayat Zagzoog, Kawthar A. Mohamed, Hye Ji J. Kim, Eunhyun D. Kim, Connor S. Frank, Tallan Black, Pramodkumar D. Jadhav, Larry A. Holbrook, Robert B. Laprairie

**Affiliations:** 1 College of Pharmacy and Nutrition, University of Saskatchewan, 3B36, Health Sciences Building, 107 Wiggins Road, Saskatoon, SK S7N 2Z4 Canada; 2 Aurora Prairie Research Centre, Aurora Cannabis, Saskatoon, SK Canada; 3 ZYUS Life Sciences, Saskatoon, SK Canada; 4 Department of Pharmacology, College of Medicine, Dalhousie University, Halifax, NS Canada

**Keywords:** Pharmacodynamics, Receptor pharmacology

## Abstract

The *Cannabis sativa* plant contains more than 120 cannabinoids. With the exceptions of ∆^9^-tetrahydrocannabinol (∆^9^-THC) and cannabidiol (CBD), comparatively little is known about the pharmacology of the less-abundant plant-derived (phyto) cannabinoids. The best-studied transducers of cannabinoid-dependent effects are type 1 and type 2 cannabinoid receptors (CB1R, CB2R). Partial agonism of CB1R by ∆^9^-THC is known to bring about the ‘high’ associated with *Cannabis* use, as well as the pain-, appetite-, and anxiety-modulating effects that are potentially therapeutic*.* CB2R activation by certain cannabinoids has been associated with anti-inflammatory activities. We assessed the activity of 8 phytocannabinoids at human CB1R, and CB2R in Chinese hamster ovary (CHO) cells stably expressing these receptors and in C57BL/6 mice in an attempt to better understand their pharmacodynamics. Specifically, ∆^9^-THC, ∆^9^-tetrahydrocannabinolic acid (∆^9^-THCa), ∆^9^-tetrahydrocannabivarin (THCV), CBD, cannabidiolic acid (CBDa), cannabidivarin (CBDV), cannabigerol (CBG), and cannabichromene (CBC) were evaluated. Compounds were assessed for their affinity to receptors, ability to inhibit cAMP accumulation, βarrestin2 recruitment, receptor selectivity, and ligand bias in cell culture; and cataleptic, hypothermic, anti-nociceptive, hypolocomotive, and anxiolytic effects in mice. Our data reveal partial agonist activity for many phytocannabinoids tested at CB1R and/or CB2R, as well as in vivo responses often associated with activation of CB1R. These data build on the growing body of literature showing cannabinoid receptor-dependent pharmacology for these less-abundant phytocannabinoids and are critical in understanding the complex and interactive pharmacology of *Cannabis*-derived molecules.

## Introduction

Biologically active compounds derived from the *Cannabis sativa* plant are referred to as ‘phytocannabinoids’. The two most-thoroughly studied phytocannabinoids are ∆^9^-tetrahydrocannabinol (∆^9^-THC) and cannabidiol (CBD). Both of these compounds are being intensively studied for their utility in treating chronic and acute pain, epilepsy, anxiety, and modulating appetite, and their potential toxicities and side effects^1^. The effects of phytocannabinoids in the human body are thought to be mediated by many different receptors including the type 1 and type 2 cannabinoid receptors (CB1R and CB2R, respectively); as well as other G protein-coupled receptors (GPCRs) such as serotonin (5HT) receptors, orphan GPCRs (e.g. GPR18, GPR55, and GPR119) and ligand-gated ion channels. The pharmacology of ∆^9^-THC is relatively well-established: THC is a CB1R and CB2R partial agonist^1–5^. The pharmacology of CBD is far-less clear. CBD has been described as a CB1R negative allosteric modulator, CB2R antagonist, GPR18 agonist, GPR55 antagonist, among other effects^6–16^. Beyond ∆^9^-THC and CBD, there are thought to be at least 120 other phytocannabinoids found in *Cannabis* for which the receptor-mediated mechanisms are still actively being investigated^2–5^. Examples of these lesser-known phytocannabinoids include ∆^9^-tetrahydrocannabidiolic acid (∆^9^-THCa), tetrahydrocannabivarin (THCV), cannabigerol (CBG), cannabichromene (CBC), and cannabidivarin (CBDV)^2–5^.

CB1R and CB2R, their endogenous agonists 2-arachidonoylglycerol (2-AG) and anandamide (AEA), and the associated anabolic and catabolic enzymes constitute the endocannabinoid system^1,5^. CB1R activation inhibits nociception and locomotor activity, activates reward pathways, and regulates mood, memory and cognition, and central hormone homeostasis^1^. CB2R activation inhibits the inflammatory response in lymphocytes and microglia^1^. Interest in the development of compounds that target CB1R and CB2R is at an all-time high because of their multiple physiological roles and consequent associations with many different disease states. To date, this interest has focused mainly on synthetic compounds that target CB1R such as antagonists/inverse agonists (e.g*.* rimonabant), negative allosteric modulators (e.g. Org27569), and positive allosteric modulators (e.g. GAT211 and ZCZ011); as well as inhibitors of cannabinoid catabolic enzymes that increase 2-AG or AEA levels^17,18^. Comparatively, little research has been done to isolate and characterize the wide variety of naturally-occurring cannabinoids of *Cannabis* (i.e. phytocannabinoids)^2,19^. The work that has been done indicates that many of these ligands are active at CB1R, CB2R, and other receptors^2,19^. Within the last three years, an influx of high-quality studies has examined the pharmacology of subsets of these phytocannabinoids for their specific activity, receptor affinity, ligand bias, and neuroprotective properties in rodent models^19–22^. Previously, our group has characterized the pharmacology of endogenous, synthetic, and plant-derived cannabinoids in vitro and in vivo^10,11,15,23,24^. In the present study, we utilized high- and medium-throughput assay systems to screen phytocannabinoids against one another and a reference compound, CP55,940, to directly assess their pharmacodynamic activity.

**Figure 1:**
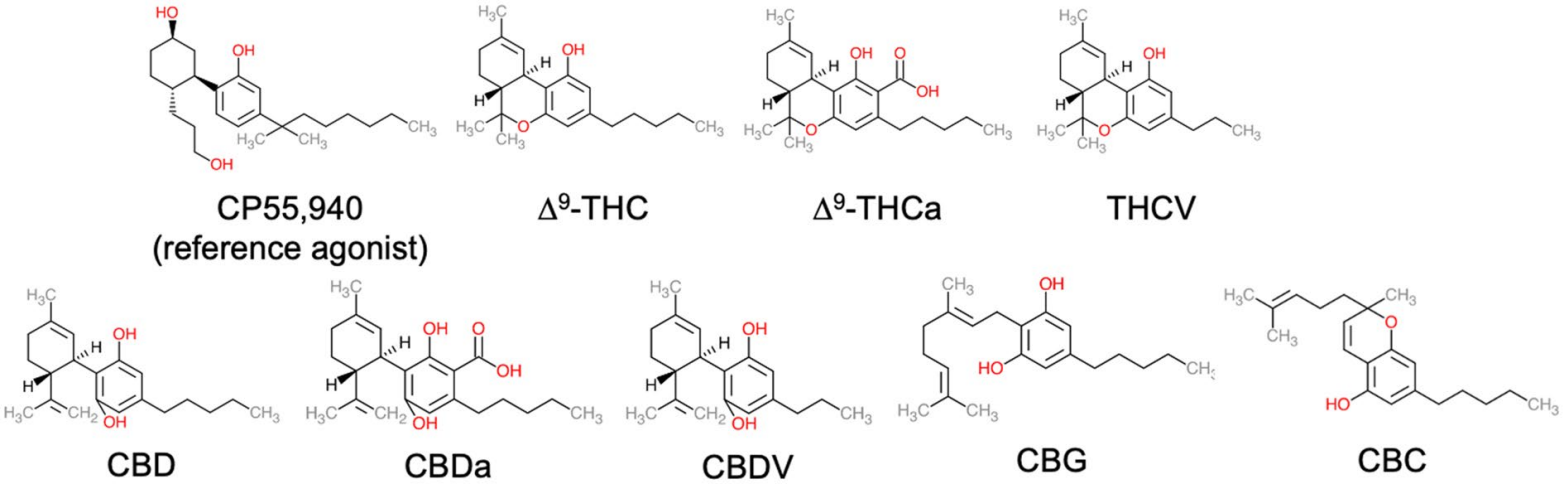
Cannabinoids assessed in this study. Chemical structures were drawn in Microsoft PowerPoint by the authors.

The primary aim of the present study was to explore the CB1R-dependent, CB2R-dependent, and in vivo pharmacology of 8 phytocannabinoids: ∆^9^-THC, ∆^9^-THCa, THCV, CBD, cannabidiolic acid (CBDa), CBDV, CBG, and CBC (Fig. 1). All compounds were assayed for the displacement of [^3^H]CP55,940, inhibition of forskolin-stimulated cAMP accumulation, and βarrestin2 recruitment in Chinese hamster ovary (CHO) cells stably expressing either human CB1R or CB2R. Compounds were assessed for signaling bias between inhibition of cAMP and βarrestin2 and selectivity between the two cannabinoid receptors. Compounds were further screened in the in vivo tetrad assays for cataleptic, hypothermic, anti-nociceptive, locomotive, and anxiety-modifying activities. We observed that all compounds tested displayed some degree of activity at CB1R or CB2R, with several being weak partial agonists. As Canada and other jurisdictions increasingly permit the use of *Cannabis* for medicinal and non-medicinal purposes, this research provides critical insight about the therapeutic potential and utility of phytocannabinoids.

## Results

### Type 1 cannabinoid receptor (CB1R)

#### Radioligand binding

**Figure 2:**
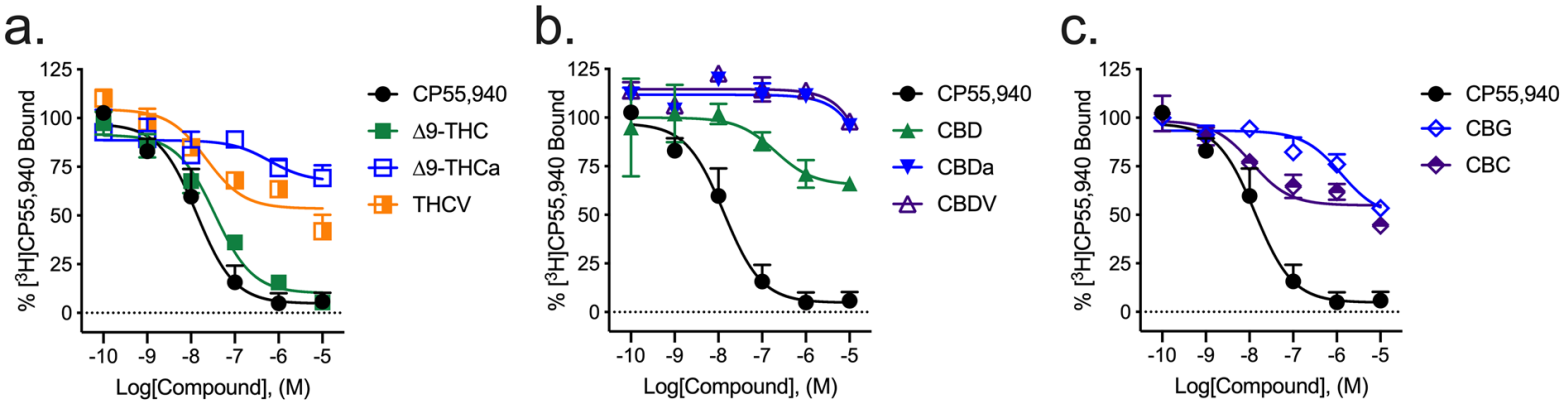
[^3^H]CP55,940 displacement from hCB1R CHO cell membranes. Compound activity was quantified for [^3^H]CP55,940 binding in CHO cells stably expressing hCB1R and treated with 0.1 nM–10 µM (**a**) THC-like compounds; (**b**) CBD-like compounds; or (**c**) CBG, or CBC. Data were fit to a variable slope (4 parameter) non-linear regression in GraphPad (v. 8). n ≥ 6 independent experiments performed in duplicate. Data are expressed as mean ± SEM. *K*_i_ and *E*_min_ are reported in Table 1.

Most cannabinoids tested displayed some ability to displace [^3^H]CP55,940 from hCB1R. Only ∆^9^-THC fully displaced [^3^H]CP55,940 from hCB1R within the concentration range tested (Fig. 2a; Table 1). ∆^9^-THCa, THCV, and CBC partially displaced [^3^H]CP55,940 from hCB1R within the concentration range tested, indicating binding at distinct or incompletely overlapping sites (i.e., non-competitive binding) (Fig. 2a,c; Table 1). CBD, CBDa, CBDV, and CBG weakly displaced [^3^H]CP55,940 at concentrations ≥ 1 µM (Fig. 2b; Table 1). The estimated binding affinities of ∆^9^-THCa and CBG were significantly lower than that of the reference compound, CP55,940, and ∆^9^-THC (Table 1). Some compounds, such as THCV and CBC, displayed comparatively wide confidence intervals around the estimated *K*_i_ values (Table 1). These values may be the result of complex pharmacology for these compounds and their incomplete competition with [^3^H]CP55,940 resulting in an inherently ‘noisy’ system. These data stress the importance of multiplicity of approach (here inhibition of cAMP accumulation, βarrestin2 recruitment, and in vivo studies) for a more comprehensive understanding of a compound’s activity. The estimated binding affinities of several compounds were not estimated if the upper and lower bounds of the competition binding curve could not be seen within the concentration range used, these compounds *K*_i_ are listed as > 10,000 in Table 1.

**Table 1:** [^3^H]CP55,940 displacement from hCB1R or hCB2R CHO cell membranes.

Compound	hCB1R		hCB2R	
	*K*_i_ (nM)	*E*_min_ (%)	*K*_i_ (nM)	*E*_min_ (%)
CP55,940	13 (5.6–33)	0.0 ± 5.2	29 (13–67)	0.0 ± 6.2
∆^9^-THC	36 (17–62)	10 ± 3.5	31 (15–62)	7.4 ± 4.4
∆^9^-THCa	620 (180–970)*^	67 ± 11*^	1.3 (0.33–6.3)*^†	49 ± 7.2*^
THCV	22 (5.0–140)	54 ± 4.1*^	47 (21–270)	27 ± 9.5
CBD	200 (140–370)^	65 ± 11*^	240 (24–560)	54 ± 9.3*^
CBDa	> 10,000	96 ± 6.5*^	12 (4.9–77)	30 ± 7.8^†^
CBDV	> 10,000	96 ± 5.1*^	140 (96–280)*^	22 ± 9.3^†^
CBG	1300 (520–8400)*^	49 ± 7.2*^	490 (130–2500)*^	32 ± 8.1
CBC	11 (1.9–91)	55 ± 3.7*^	27 (8.9–83)	9.7 ± 5.9^†^

Compound activity was quantified for [^3^H]CP55,940 binding in CHO cells stably expressing hCB1R or hCB2R and treated with compounds. Data were fit to a variable slope (4 parameter) non-linear regression in GraphPad (v. 8). n ≥ 6 independent experiments performed in duplicate. *E*_Min_ refers to the bottom of the concentration–response curve. Data are expressed as nM with 95% CI or %CP55,940 response, mean ± SEM. *p < 0.05 compared to CP55,940 within receptor; ^p < 0.05 compared to ∆^9^-THC within receptor; †p < 0.05 between receptors; as determined via non-overlapping 95% CI or one-way ANOVA followed by Tukey's post-hoc test. Corresponding graphs are presented in Figs. 2 and 5.

#### Inhibition of forskolin-stimulated cAMP

**Figure 3:**
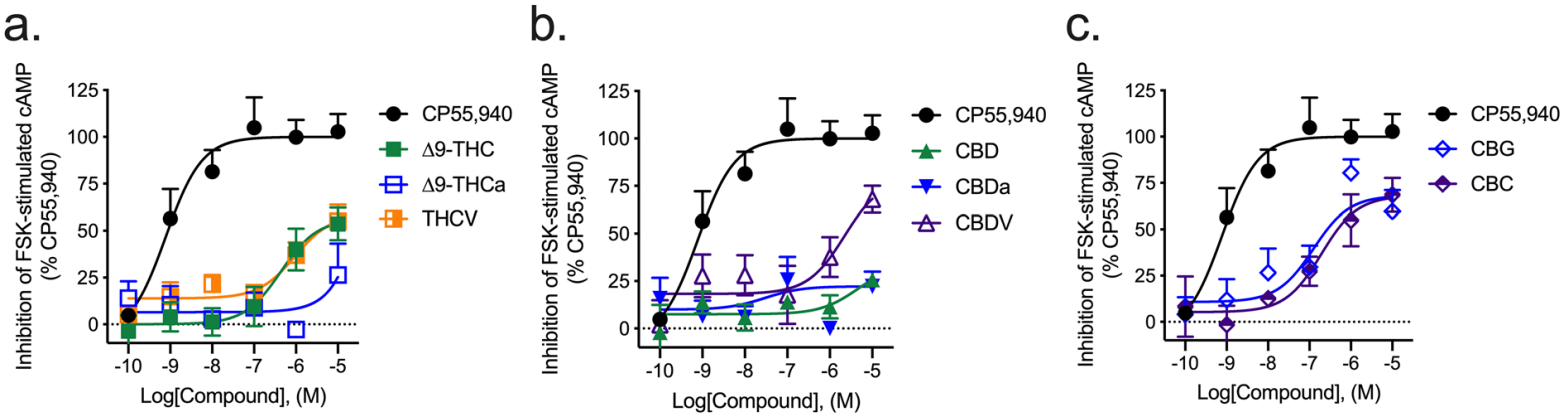
hCB1R-dependent inhibition of FSK-stimulated cAMP accumulation. hCB1R-dependent inhibition of FSK-stimulated cAMP accumulation was quantified in HitHunter CHO cells stably expressing hCB1R and treated with 0.1 nM–10 µM (**a**) THC-like compounds; (**b**) CBD-like compounds; or (**c**) CBG, or CBC for 90 min. Data were fit to a variable slope (4 parameter) non-linear regression in GraphPad (v. 8). n ≥ 6 independent experiments performed in triplicate. *E*_Max_ refers to the top of the concentration–response curve. Data are expressed as mean ± SEM. EC_50_ and *E*_max_ are reported in Table 2.

The majority of compounds tested for their ability to inhibit the accumulation of forskolin (FSK)-stimulated cAMP were partial agonists of this effect at hCB1R. All compounds displayed an *E*_max_ in the cAMP inhibition assay that was less than that of CP55,940 (Fig. 3; Table 2). ∆^9^-THCa, CBD, and CBDa were less efficacious than ∆^9^-THC (Fig. 3; Table 2). ∆^9^-THC, THCV, and CBC were less potent than the reference agonist CP55,940 (Table 2).

**Table 2:** hCB1R- or hCB2R-dependent inhibition of FSK-stimulated cAMP accumulation.

Compound	hCB1R		hCB2R	
	EC_50_ (nM)	*E*_max_ (%)	EC_50_ (nM)	*E*_max_ (%)
CP55,940	7.7 (0.13–14)	100 ± 6.2	4.0 (0.86–12)	100 ± 8.2
∆^9^-THC	240 (100–320)*	56 ± 9.6*	18.0 (5.0–102)	76 ± 8.6*
∆^9^-THCa	> 10,000	35 ± 11	1800 (360–3800)*^	95 ± 1.5^^†^
THCV	260 (46–1200)*	59 ± 3.9*	280 (49–610)*^	79 ± 1.3*^†^
CBD	> 10,000	26 ± 1.6*	> 10,000	61 ± 2.3*^†^
CBDa	30 (2.8–200)	22 ± 1.1*	140 (29–310)*	32 ± 4.2*^†^
CBDV	> 10,000	68 ± 5.7*	5.0 (0.46–33)	51 ± 12*
CBG	120 (7.4–700)	68 ± 2.4*	130 (30–550)*	39 ± 11*^^†^
CBC	190 (23–1700)*	68 ± 9.7*	7.1 (2.0–24)	76 ± 5.4*

hCB1R- or hCB2R-dependent inhibition of FSK-stimulated cAMP accumulation was quantified in HitHunter CHO cells stably expressing hCB1R or hCB2R and treated with compounds for 90 min. Data were fit to a variable slope (4 parameter) non-linear regression in GraphPad (v. 8). n ≥ 6 independent experiments performed in triplicate. *E*_Max_ refers to the top of the concentration–response curve. Data are expressed as nM with 95% CI or %CP55,940 response, mean ± SEM. *p < 0.05 compared to CP55,940 within receptor; ^p < 0.05 compared to ∆^9^-THC within receptor; ^†^p < 0.05 compared to hCB1R within compound; as determined via non-overlapping 95% CI or one-way ANOVA followed by Tukey's post-hoc test. Corresponding graphs are presented in Figs. 3 and 6.

#### βarrestin2 recruitment

**Figure 4:**
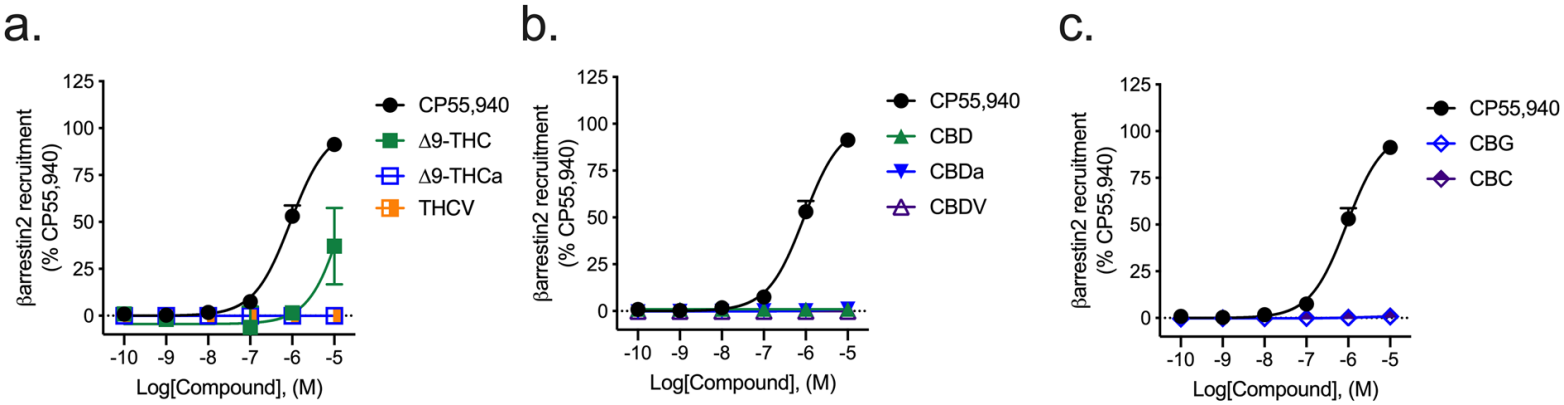
hCB1R-dependent recruitment of βarrestin2. hCB1R-dependent recruitment of βarrestin2 was quantified in PathHunter CHO cells stably expressing hCB1R and treated with 0.1 nM–10 µM (**a**) THC-like compounds; (**b**) CBD-like compounds; or (**c**) CBG, or CBC for 90 min. Data were fit to a variable slope (4 parameter) non-linear regression in GraphPad (v. 8). n ≥ 6 independent experiments performed in triplicate. *E*_Max_ refers to the top of the concentration–response curve. Data are expressed as mean ± SEM. EC_50_ and *E*_max_ are reported in Table 3.

All phytocannabinoids tested displayed little-to-no activity in the βarrestin2 recruitment assay relative to the reference agonist CP55,940 and within the concentration range used (Fig. 4). Among the phytocannabinoids, ∆^9^-THC was the most-efficacious displaying an *E*_max_ value of 37 ± 7.5% relative to CP55,940 (Fig. 4; Table 3).

**Table 3:** hCB1R- or hCB2R-dependent recruitment of βarrestin2.

Compound	hCB1R		hCB2R	
	EC_50_ (nM)	*E*_max_ (%)	EC_50_ (nM)	*E*_max_ (%)
CP55,940	920 (700–1200)	100 ± 5.6	560 (410–760)	100 ± 3.4
∆^9^-THC	> 10,000	37 ± 7.5*	94 (78–210)*	46 ± 4.9*
∆^9^-THCa	> 10,000	4.1 ± 1.5*^	> 10,000	15 ± 2.5*^^†^
THCV	> 10,000	0.05 ± 2.1*^	> 10,000	50 ± 6.8*^†^
CBD	> 10,000	1.0 ± 1.6*^	58 (44–74)*^	23 ± 2.8*^†^
CBDa	> 10,000	0.10 ± 0.11*^	> 10,000	18 ± 2.5*^^†^
CBDV	> 10,000	0.96 ± 0.75*^	> 10,000	64 ± 13*^†^
CBG	> 10,000	0.41 ± 0.45*^	> 10,000	22 ± 1.2*^^†^
CBC	> 10,000	6.9 ± 0.96*^	> 10,000	12 ± 2.8*^^^

hCB1R-or hCB2R-dependent recruitment of βarrestin2 was quantified in PathHunter CHO cells stably expressing hCB1R or hCB2R and treated with compounds for 90 min. Data were fit to a variable slope (4 parameter) non-linear regression in GraphPad (v. 8). n ≥ 6 independent experiments performed in triplicate. *E*_Max_ refers to the top of the concentration–response curve. Data are expressed as nM with 95% CI or %CP55,940 response, mean ± SEM. *p < 0.05 compared to CP55,940; ^p < 0.05 compared to ∆^9^-THC within assay and measurement; †p < 0.05 compared to hCB1R within compound; as determined via non-overlapping 95% CI or one-way ANOVA followed by Tukey's post-hoc test. Corresponding graphs are presented in Figs. 4 and 7.

### Type 2 cannabinoid receptor (CB2R)

#### Radioligand binding

**Figure 5:**
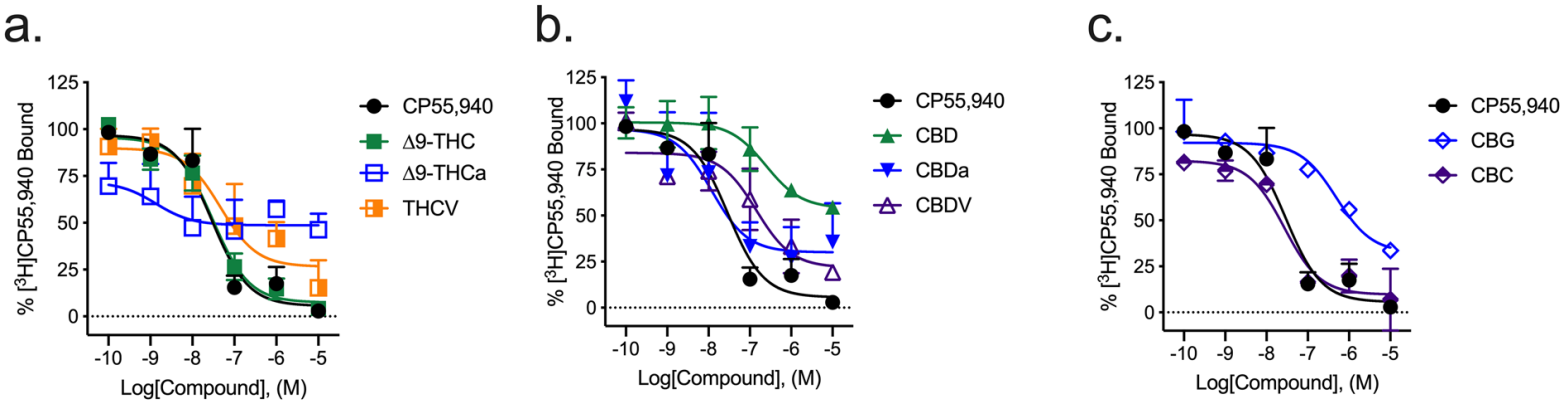
[^3^H]CP55,940 displacement from hCB2R CHO cell membranes. Compound activity was quantified for [^3^H]CP55,940 binding in CHO cells stably expressing hCB2R and treated with 0.1 nM–10 µM (**a**) THC-like compounds; (**b**) CBD-like compounds; or (**c**) CBG, or CBC. Data were fit to a variable slope (4 parameter) non-linear regression in GraphPad (v. 8). n ≥ 6 independent experiments performed in duplicate. Data are expressed as mean ± SEM. *K*_i_ and *E*_min_ are reported in Table 1.

Most cannabinoids tested displaced [^3^H]CP55,940 from hCB2R. ∆^9^-THC, THCV, CBDa, CBDV, CBG, and CBC displaced [^3^H]CP55,940 from hCB2R to an extent that was not different from CP55,940 (Fig. 5; Table 1). ∆^9^-THCa, THCV, and CBD partially displaced [^3^H]CP55,940 from hCB2R within the concentration range tested, indicating binding at distinct or incompletely overlapping sites (i.e., non-competitive binding) (Fig. 5; Table 1). The estimated binding affinity of CBDV, CBG, and CBC was significantly lower than that of the reference compound, CP55,940, and ∆^9^-THC (Table 1).

#### Inhibition of forskolin-stimulated cAMP

**Figure 6:**
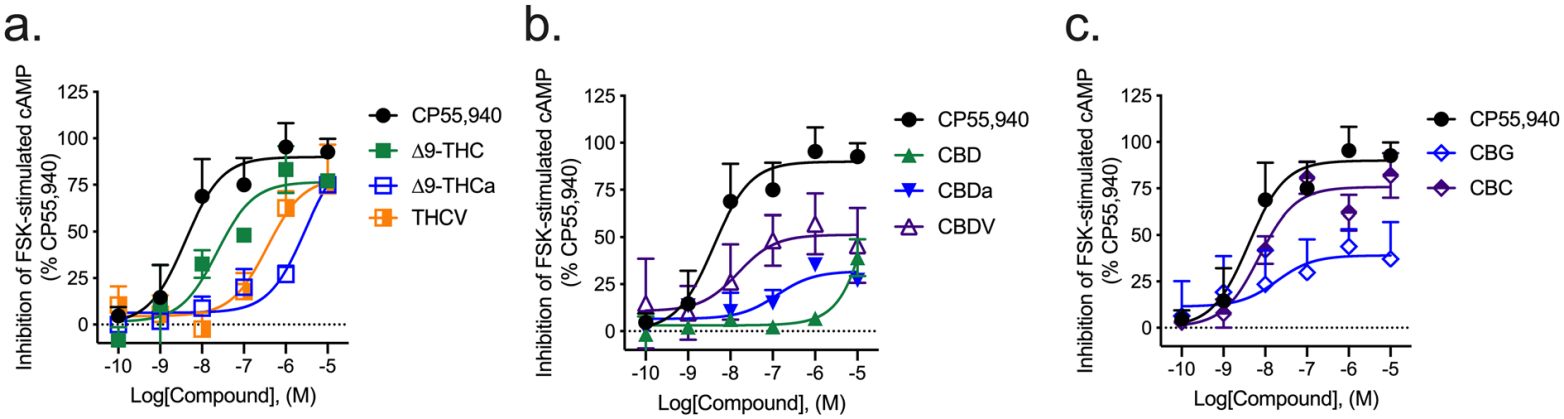
hCB2R-dependent inhibition of FSK-stimulated cAMP accumulation. hCB2R-dependent inhibition of FSK-stimulated cAMP accumulation was quantified in HitHunter CHO cells stably expressing hCB2R and treated with 0.1 nM–10 µM (**a**) THC-like compounds; (**b**) CBD-like compounds; or (**c**) CBG, or CBC for 90 min. Data were fit to a variable slope (4 parameter) non-linear regression in GraphPad (v. 8). n ≥ 6 independent experiments performed in triplicate. *E*_Max_ refers to the top of the concentration–response curve. Data are expressed as mean ± SEM. EC_50_ and *E*_max_ are reported in Table 2.

The cannabinoids tested here were agonists or partial agonists of inhibition of cAMP accumulation at hCB2R. ∆^9^-THCa, THCV, CBDa, and CBG were less potent inhibitors of cAMP accumulation than CP55,940 (Fig. 6; Table 2). ∆^9^-THCa and THCV were also less potent inhibitors of cAMP accumulation than ∆^9^-THC (Fig. 6; Table 2). ∆^9^-THC, THCV, CBDa, CBDV, CBG, and CBC were less efficacious ligands (i.e., partial agonists) for CB2R-mediated inhibition of cAMP than CP55,940 (Fig. 6; Table 2). ∆^9^-THCa was a more efficacious ligand for CB2R-mediated inhibition of cAMP than ∆^9^-THC, whereas CBDa and CBG were less efficacious ligands for CB2R-mediated inhibition of cAMP than ∆^9^-THC (Fig. 6; Table 2).

#### βarrestin2 recruitment

**Figure 7:**
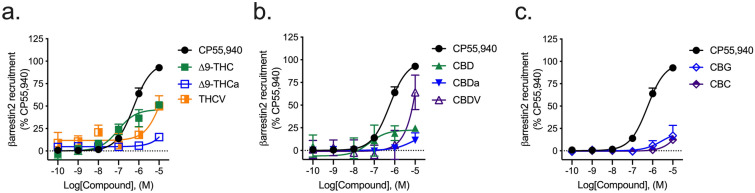
hCB2R-dependent recruitment of βarrestin2. hCB2R-dependent recruitment of βarrestin2 was quantified in PathHunter CHO cells stably expressing hCB2R and treated with 0.1 nM–10 µM (**a**) THC-like compounds; (**b**) CBD-like compounds; or (**c**) CBG, or CBC for 90 min. Data were fit to a variable slope (4 parameter) non-linear regression in GraphPad (v. 8). n ≥ 6 independent experiments performed in triplicate. *E*_Max_ refers to the top of the concentration–response curve. Data are expressed as mean ± SEM. EC_50_ and *E*_max_ are reported in Table 3.

∆^9^-THC and CBD displayed modest agonist activity at hCB2R in the βarrestin2 recruitment assay relative to the reference agonist CP55,940 and within the concentration range used (Fig. 7). THCV and CBDV treatment produced demonstrable efficacy at hCB2R in the βarrestin2 recruitment assay, but with very low potency (Fig. 7; Table 3).

### Ligand bias

**Figure 8:**
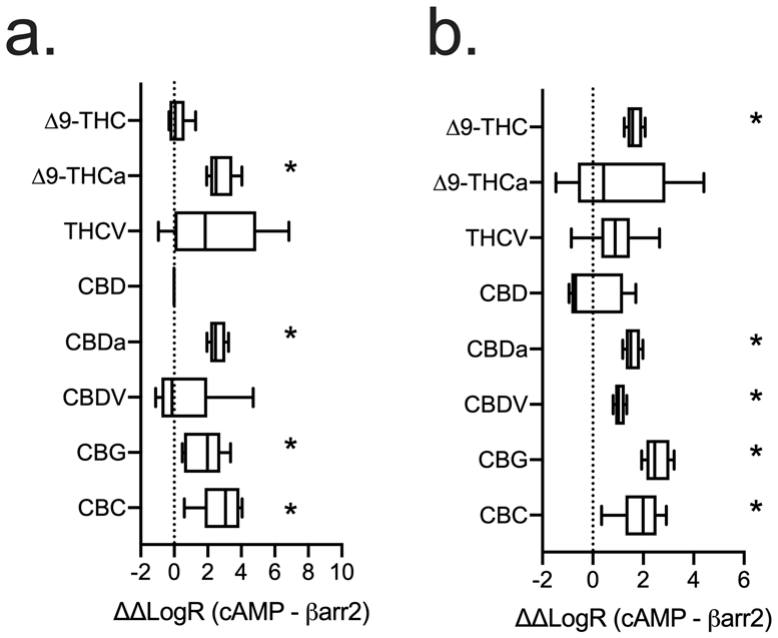
Analysis of ligand bias for phytocannabinoids at hCB1R (**a**) and hCB2R (**b**). Data from cAMP inhibition assays (Figs. 2, 5) and βarrestin2 recruitment assays (Figs. 3, 6) were fit to the operational model of Black and Leff (1983) using CP55,940 as the reference agonist and bias factor—reported here as ∆∆LogR—was calculated for each ligand between inhibition of FSK-stimulated cAMP accumulation and βarresitn2 recruitment. n ≥ 6 independent experiments performed in triplicate. Data are expressed as mean ± 95% CI (box) and minimum and maximum observed values (whiskers). *p < 0.05 relative to 0 as determined by non-overlapping 95% CI.

Phytocannabinoid ligand bias at hCB1R and hCB2R was assessed between inhibition of FSK-stimulated cAMP accumulation and βarrestin2 recruitment using the operational model of Black and Leff and as described previously^11,25^. Analyses were conducted such that ∆∆LogR values > 0 represented bias for inhibition of cAMP and ∆∆LogR values < 0 represented bias for the recruitment of βarrestin2 (Fig. 8). From these analyses, we observed that, at hCB1R, ∆^9^-THCa, CBDa, CBG, and CBC displayed a bias for the inhibition of cAMP relative to βarrestin2 (Fig. 8a). No phytocannabinoids tested displayed βarrestin2 recruitment bias at hCB1R. At hCB2R, ∆^9^-THC, CBDa, CBDV, CBG, and CBC all displayed a bias for inhibition of cAMP relative to βarrestin2; and no phytocannabinoids displayed a bias for βarrestin2 recruitment (Fig. 8b). Of note, CBDa, CBG, and CBC displayed a bias for inhibition of cAMP at both hCB1R and hCB2R (Fig. 8). Other phytocannabinoids tested did not display a bias for either inhibition of cAMP or βarrestin2 recruitment at hCB1R or hCB2R.

### Selectivity between hCB1R and hCB2R

We estimated the selectivity of the phytocannabinoids tested by comparing between results obtained with hCB1R or hCB2R within each assay.

#### Non-selective

∆^9^-THC did not display selectivity between hCB1R and hCB2R in the assays tested here, and in keeping with previous reports^3,26^. CBD did not display consistent selectivity because it displayed greater efficacy for inhibition of cAMP at hCB2R and measurable potency for βarrestin2 hCB2R, but greater efficacy at hCB1R in the βarrestin2 recruitment assay (Table 3). CBG’s displacement of [^3^H]CP55,940 from hCB1R and hCB2R was not different, nor was its potency to inhibition cAMP accumulation at either receptor although CBG displayed lower efficacy to inhibit cAMP accumulation at hCB2R compared to hCB1R (Tables 1,2). In contrast, CBG had measurable potency and greater efficacy for βarrestin2 recruitment at hCB2R relative to hCB1R (Tables 3). No phytocannabinoids tested here displayed consistent hCB1R selectivity.

#### hCB2R-selective

The majority of phytocannabinoids tested here displayed consistent selectivity for hCB2R relative to hCB1R across the three assays used. ∆^9^-THCa was hCB2R-selective because it displayed a greater affinity for hCB2R, displaced [^3^H]CP55,940 to a greater extent at hCB2R, had measurable potency and greater efficacy for cAMP inhibition at hCB2R, and greater efficacy for βarrestin2 recruitment at hCB2R than at hCB1R for any of the aforementioned assays (Tables 1, 2, 3). THCV displayed greater efficacy for inhibition of cAMP and βarrestin2 recruitment at hCB2R than hCB1R (Tables 2, 3). CBDa displayed displaced [^3^H]CP55,940 to a greater extent at hCB2R and had greater efficacy for cAMP inhibition and βarrestin2 recruitment at hCB2R than at hCB1R (Tables 1, 2, 3). CBDV displaced [^3^H]CP55,940 to a greater extent at hCB2R, had measurable potency and equivalent efficacy for cAMP inhibition at hCB2R, and greater efficacy for βarrestin2 recruitment at hCB2R than at hCB1R (Tables 1, 2, 3). Finally, CBC displaced [^3^H]CP55,940 to a greater extent at hCB2R than hCB1R (Table 1).

### Tetrad analyses

**Figure 9:**
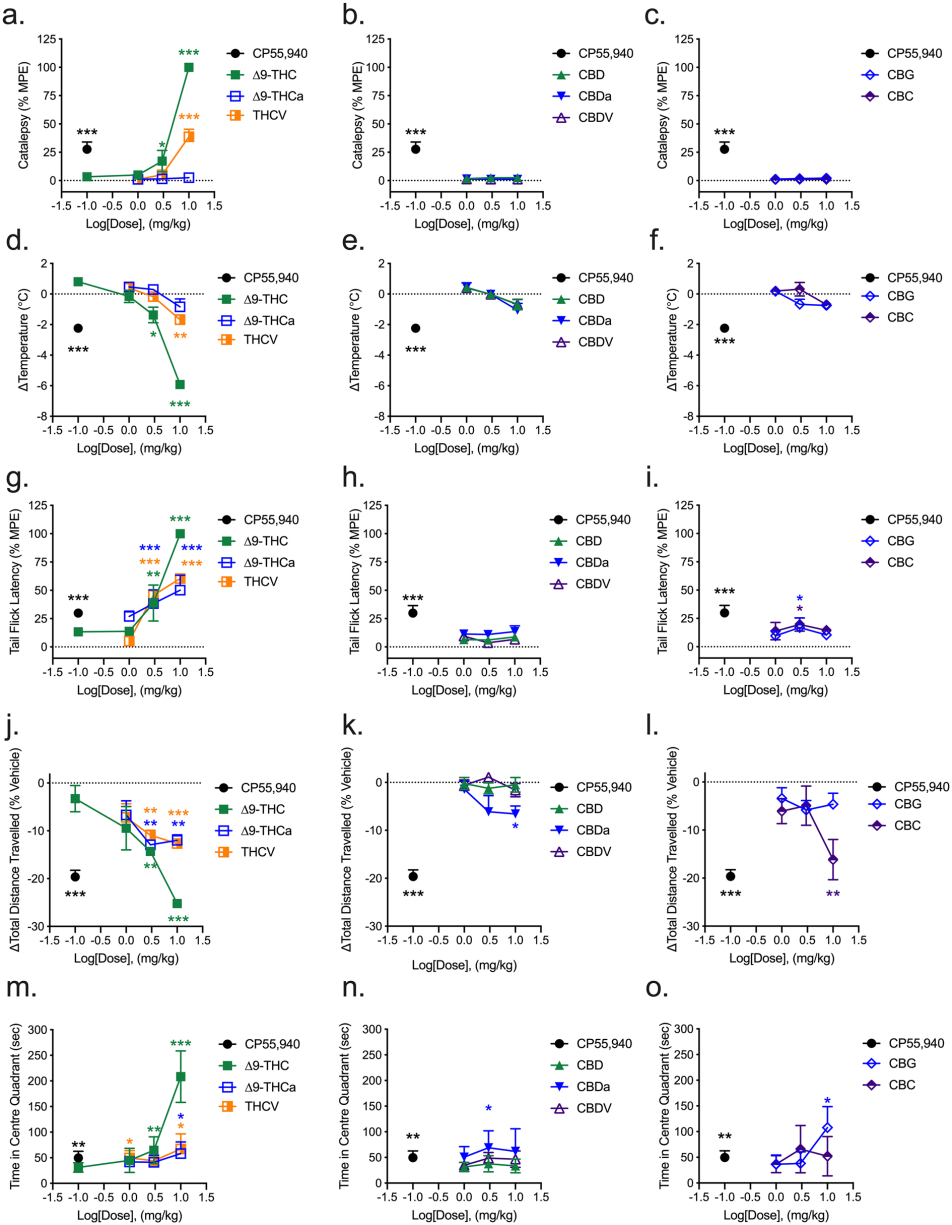
Acute tetrad effects in male C57BL/6 mice. Male mice aged 6–12 weeks were treated with 0.1–10 mg/kg *i.p.* of phytocannabinoids and assessed for catalepsy (**a**–**c**, 5 min post-injection), body temperature (**d**–**f**, 10 min post-injection), nociception in the tail flick assay (**g**–**i**, 15 min post-injection), and both locomotion (**j**–**l**) and time in the centre quadrant (**m**–**o**) in the OFT 1 h post-injection. THC-like compounds are shown in panels **a**, **d**, **g**, **j**, and **m**. CBD-like compounds are shown in panels **b**, **e**, **h**, **k**, and **n**. CBG, and CBC are shown in panels **c**, **f**, **i**, **l**, and **o**. (**a**–**c**) Catalepsy data are expressed as the % maximum possible effect (MPE, i.e*.* 60 s). (**d**–**f**) Body temperature data are expressed as change (∆) from baseline (°C). (**g**–**i**) Tail flick latency data are expressed as the % maximum possible effect (MPE, i.e*.* 20 s). (**j**–**l**) Locomotion data are expressed as % change from baseline total distance travelled. (**m**–**o**) Time in centre quadrant data are expressed as sec during 5 min OFT trials. n = 6/treatment. All data are expressed as mean ± SEM. *p < 0.05, **p < 0.01, ***p < 0.001 relative to vehicle for each assay as determined via one-way ANOVA followed by Tukey’s post-hoc analyses. Asterisk colour matches the treatment group assessed.

Male C57Bl/6 mice aged 6–12 weeks were treated with 0.1–10 mg/kg of phytocannabinoids, or 0.1 mg/kg CP55,940 as a reference agonist, and assessed for catalepsy, hypothermia, nociception, locomotion, and anxiety-like behaviours following injection. CP55,940 (0.1 mg/kg) treatment produced a cataleptic response in the ring holding assay at 25% of the maximum possible effect (MPE; 60 s = 100% in the ring holding assay). ∆^9^-THC and THCV also evoked cataleptic responses at 3 (17%) and 10 mg/kg (100%) and 10 mg/kg (39%), respectively (Fig. 9a). No other tested phytocannabinoid evoked a cataleptic response within the dose range tested (Fig. 9a–c). Similar to the results observed in the ring holding assay, only CP55,940 (0.1 mg/kg), ∆^9^-THC (3 and 10 mg/kg), and THCV (10 mg/kg) decreased body temperature in mice; all other phytocannabinoids tested failed to elicit a hypothermic response at the doses tested (Fig. 9d–f). In addition to CP55,940, ∆^9^-THC, ∆^9^-THCa, and THCV produced a dose-dependent increase in anti-nociceptive effects in mice (Fig. 9g). CBG and CBC also produced a weak anti-nociceptive effect at 3 mg/kg (Fig. 9i), but all other phytocannabinoids test did not alter tail flick latency (Fig. 9g–i). In the open field test (OFT) and in addition to CP55,940, ∆^9^-THC, ∆^9^-THCa, and THCV reduced total locomotion in a dose-dependent manner (Fig. 9j). Hypolocomotive responses were also observed in mice treated with 10 mg/kg CBDa and CBC (Fig. 9k,l). Finally, CP55,940 (0.1 mg/kg), ∆^9^-THC (3, 10 mg/kg), ∆^9^-THCa (10 mg/kg), THCV (1, 10 mg/kg), CBDa (3 mg/kg), and CBG (10 mg/kg) increased the time mice spent in the central quadrant of the OFT, indicative of a modelled anxiolytic effect for these compounds (Fig. 9m–o).

## Discussion

In general, we observed that all phytocannabinoids tested displayed some degree of activity at either CB1R or CB2R in cell culture assays. The incomplete competition of these phytocannabinoids with [^3^H]CP55,940 suggests that the occupied binding site of these ligands differs slightly from that of CP55,940. It is possible that the phytocannabinoids tested bound only a subset of amino acids in the CB1R ligand binding site(s) compared to CP55,940, as has been shown for CBD, Org27569, rimonabant, and anandamide^10,18,27,28^.

∆^9^-THC that is commonly consumed in cannabis products is a decarboxylated derivative of the naturally present ∆^9^-THCa^19^. In this study, ∆^9^-THCa partially displaced [^3^H]CP55,940 from CB1R and CB2R, with greater affinity for CB2R; and was a weak partial agonist of CB1R-dependent inhibition of cAMP accumulation and a weakly potent agonist of CB2R-dependent cAMP accumulation. ∆^9^-THCa produced anti-nociceptive and hypolocomotive effects at 3 and 10 mg/kg, and anxiolytic-like effects at 10 mg/kg. ∆^9^-THCa has previously been shown to bind murine cannabinoid receptors weakly^21^. Similar to our findings, Palomarés et al.^29^ recently reported ∆^9^-THCa binding to CB1R and CB2R, with potential orthosteric and allosteric activity at CB1R. In vivo, the anti-inflammatory activity of ∆^9^-THCa in a rodent model of arthritis was CB1R- and peroxisome proliferator-activated receptor γ (PPARγ)-dependent^29^. Similar neuroprotective and PPAR-dependent effects have been observed in rodent models of Huntington’s disease^30^. Here, ∆^9^-THCa displayed similar efficacy to ∆^9^-THC in the cAMP inhibition assay but a lower affinity for CB1R in [^3^H]CP55,940 competition experiments. These data corroborate the findings of Palomarés et al.^29^ and indicate the antinociceptive effect of ∆^9^-THCa observed here is likely in part due to CB1R agonism. Our data, together with previous studies, indicate ∆^9^-THCa may mediate neuroprotective and anti-inflammatory actions via CB1R, CB2R, and PPARγ when administered at sufficiently high concentrations and in the absence of other cannabinoids.

THCV has been posited to act at both CB1R and CB2R, and is considered by some to behave as a CB1R antagonist and CB2R agonist^31–33^; reviewed in^19^. Here, we found that THCV was able to displace [^3^H]CP55,940 from both CB1R and CB2R. Unlike previous studies suggesting THCV is a CB1R antagonist, THCV did produce a weak partial agonist response in the CB1R cAMP inhibition assay. THCV was an agonist of CB2R-dependent cAMP inhibition, as described previously^34^ and βarrestin2 recruitment. In vivo, THCV produced cataleptic (10 mg/kg), hypothermic (10 mg/kg), anti-nociceptive (3 and 10 mg/kg), hypolocomotive (3 and 10 mg/kg), and anxiolytic (3 and 10 mg/kg) effects. These effects are consistent with others’ observations of anti-epileptic, hypolocomotive, neuroprotective effects in the range of 0.25–2.5 mg/kg^32,33^; reviewed in^19^. Bolognini et al.^34^ demonstrated that THCV is able to reduce hyperalgesia in mice via both CB1R and CB2R, because anti-hyperalgesic effects were limited by both the CB1R antagonist SR141716A and the CB2R antagonist SR144528. Given the in vitro efficacy of THCV at both CB1R and CB2R, it is likely that the in vivo effects of THCV observed here are dependent on both cannabinoid receptors, as has been described previously^34^.

CBD has been much more-extensively studied than the other phytocannabinoids. CBD has been described as a CB1R negative allosteric modulator^10,14,15,29^; CB2R antagonist^35^; GPR18, GPR55, and 5HT3A antagonist ^1,36^; 5HT1A, 5HT2A, adenosine 1A, and PPARγ partial agonist^16,37–39^; and an allosteric modulator of the µ- and δ-opioid receptors^4,9^. In this study, we included CBD in an assay format that would detect agonism at CB1R and CB2R. Not surprisingly, CBD displayed low affinity at both CB1R and CB2R and displayed minimal activity at both receptors. Similarly, we observed no effects of CBD between 1 and 10 mg/kg in the tetrad of assays, as expected. CBD has been shown in animal models of disease to have anti-convulsant, anti-inflammatory, and anti-nociceptive effects; all of which appear to be independent of CB1R and CB2R^19^.

CBDa was inactive at CB1R and displayed weak partial agonism at CB2R in our cell culture assays. Navarro et al.^12^ reported higher than anticipated affinity and activity of CBDa in both [^3^H]CP55,940 binding for CB1R and CB2R in CHO cells and signaling in HEK-293T cells, which were similar to our findings. In vivo, CBDa produced significant hypolocomotive and anxiolytic effects in the OFT. Beyond CB1R and CB2R, CBDa has shown efficacy in reducing inflammatory pain and nausea^40,41^, seizure incidence^42^, and Parkinsonian signs in rodent models^13^. Accumulating data suggest the in vivo effects of CBDa are 5HT1A-mediated^40^. Therefore, the effects observed in this study in the tetrad maybe 5HT1A-mediated, rather than cannabinoid receptor-dependent.

CBDV displayed little to no activity at CB1R but did display demonstrable affinity and activity at CB2R. Similarly, Navarro et al.^12^ observed that CBDV was nearly inactive at CB1R with greater activity at CB2R. However, the binding affinity of CBDV to CB2R was greater in our experiments compared to those of Navarro et al.^12^. This difference may be due to differing radioligand concentrations and/or differential expression of the receptor in our cell models. In our assays, CBDV displayed a bias toward the inhibition of cAMP relative to βarrestin2 recruitment; whereas previous reports in HEK-293T cells have described the opposite, although these bias analyses were conducted by different methods^12^. CBDV produced no significant in vivo responses in our assays. Earlier reports have shown anti-convulsant effects associated with CBDV treatment in rodent models and occur at 200–400 mg/kg *p.o.*, doses higher than were tested here (reviewed in^19^).

In CHO cells stably expressing human receptors, CBG was a weak partial agonist of both CB1R- and CB2R-dependent inhibition of cAMP that displaying a low affinity for CB1R and comparatively higher affinity for CB2R. Previous reports have similarly described CBG as a weak partial agonist of these two receptors, with low (> 1 µM) affinity for both^6–8,43,44^. Interestingly, previous observations have also indicated that CBG-dependent recruitment of βarrestin via the cannabinoid receptors is less potent and efficacious than other signal transduction pathways^12,44^, similar to our findings. Although our data indicate a higher affinity for CBG at CB2R than previous reports (500 nM vs. > 1 µM), all of our other observations are in concordance with previous findings^12,44^. Whereas Navarro et al.^12^ observed CBG to be a balanced, non-biased, ligand at CB1R and CB2R in HEK-293T cells; CBG displayed a bias for the inhibition of cAMP in CHO cells stably expressing CB1R or CB2R. In vivo, CBG produced a small but statistically significant anti-nociceptive effect at 3 mg/kg and an anxiolytic-like effect in the OFT at 10 mg/kg. CBG has previously been reported to have disease-ameliorating anti-inflammatory effects in mouse models of Huntington’s disease, multiple sclerosis, Parkinson’s disease, and amyotrophic lateral sclerosis^45–49^. These effects were likely mediated by PPARγ, and not cannabinoid receptors^43,48,49^. CBG has also been shown to have in vitro activity at GPR55, 5HT1A, the α2 adrenoceptor, and several transient receptor potential (TRP) channels^6–8^. Therefore, it is possible the in vivo anti-nociceptive and anxiolytic effects observed in our study occurred via these, cannabinoid receptor-independent, mechanisms.

Similar to CBG, CBC was a partial agonist of both CB1R- and CB2R-dependent signaling, with great selectivity and potency at CB2R relative to CB1R in the assays utilized here. Previous studies have observed weak partial agonism of CBC at both CB1R and CB2R^8^. More recently, Udoh et al.^50^ reported that CBC produced CB2R-dependent membrane hyperpolarization in AtT20 cells. This effect was absent in CB1R-expressing AtT20 cells, indicating CB2R-specificity for CBC^50^. In vivo, CBC is able to increase neuronal viability via ERK phosphorylation in nestin-positive neural stem cells, but the receptor-specific mechanism has not been described for this effect^51^. CBC has also been shown to be anti-inflammatory and reduce hypermobility in a mouse model of gut inflammation, although these effects occur via TRPA1 and not cannabinoid receptors^52^. Similar to the findings of Izzo et al.^52^, CBC did produce a small, but statistically significant anti-nociceptive effect in the tail withdrawal assay and a hypolocomotive effect in the OFT; both of these effects may have occurred independently of cannabinoid receptors.

Based on these data, it is unclear whether the cannabinoids tested here bind an identical orthosteric site to that of ∆^9^-THC or CP55,940. Future experiments utilizing site-directed mutagenesis will need to assess this question directly. Moreover, the partial agonist effects displayed by these ligands suggests they may be functionally antagonistic in the presence of higher agonist concentrations and in vivo. This functional antagonism has been previously demonstrated for ∆^9^-THC itself when administered alongside full and potent CB1R agonists^10,53–56^. A growing body of literature also supports the notion that phytocannabinoids such as ∆^9^-THCa, CBD, and others may be able to occupy both orthosteric and allosteric sites with varying affinity, further complicating our understanding of the cannabinoid receptors^10,15,29^. Future work assessing the potential antagonist activity of these compounds in the presence of a full agonist such as CP55,940 will be able to better-classify the mechanisms of action for these compounds beyond what has been done here.

This work represents an initial step into the assessment of the pharmacology for a subset of *Cannabis*-derived phytomolecules at the most-thoroughly studied cannabinoid receptors, CB1R and CB2R. It is possible that if the phytocannabinoids tested here that displayed CB1R agonist and in vivo activity were present at sufficiently high concentrations in cannabis products; they may produce intoxicating effects similar to those of ∆^9^-THC. However, given the typically low content of these phytomolecules in cannabis products and their weak displacement of [^3^H]CP55,940, these compounds are all *probably* more likely to diminish ∆^9^-THC’s effects in whole organisms. Beyond CB1R, ∆^9^-THC itself is known to modulate the signaling of several proteins, including the orphan GPCR GPR55, and the TRP vanilloid 1 Ca^2+^ channel (TRPV1)^17^. Other cannabinoids, such as CBD, modulate the activity of a wide array of cannabinoid and non-cannabinoid receptors, including CB1R as a negative allosteric modulator, CB2R and 5HT1A as a partial agonist, the µ-opioid receptor, and PPARs^17^. Our in vivo observations that CBDa, CBG, and CBC (among other compounds tested) mediated changes in locomotion and time in the centre quadrant despite having little in vitro activity at CB1R, warrant further study to determine what other receptors are utilized by these ligands. Therefore, in order to comprehensively understand the poly-pharmacology of cannabinoid receptors in vivo, other receptor targets must be considered.

Finally, cannabis products contain many phytomolecules that are co-administered when *Cannabis* is consumed. This study represents an initial foray into potential between- and among-phytomolecule interactions that can be built upon gradually. Pharmacology is a reductionist approach to biochemical interactions that cannot always model the complex interactions occurring in nature. As initial characterizations of single ligands are made, more complex combinatorial testing of pharmacology can be carried out. Eventually, we hope to assess potential interactive effects of complex cannabinoid mixtures and assess pharmacodynamic—and pharmacokinetic—differences that stem from chemically distinct ligands.

## Methods

### Compounds

CP55,940 and SR141716A were purchased from Tocris Bioscience (Oakville, ON). All other cannabinoids were obtained at ≥ 98% purity from Aurora Prairie (Aurora Cannabis Inc., Saskatoon, SK). Because concern exists regarding the stability plant-derived cannabinoids, such as ∆^9^-THCa undergoing spontaneous decarboxylation, all compounds were aliquoted, stored at − 80 °C until use, and were used only once. Compounds were assessed for purity by high performance liquid chromatography with diode-array detection (HPLC-DAD) using well-described methods following both purification and 1-month storage at − 80 °C^57^. A representative chromatogram for ∆^9^-THCa is included in Supplementary Figure S1. [^3^H]CP55,940 (174.6 Ci/mmol) was obtained from PerkinElmer (Guelph, ON). All other reagents were obtained from Sigma-Aldrich (Oakville, ON) unless specifically noted. Compounds were dissolved in DMSO (final concentration of 0.1% in assay media for all assays) and added directly to the media at the concentrations and times indicated.

### Cell culture

Chinese hamster ovary (CHO)-K1 cells stably expressing human cannabinoid CB1R or CB2R were maintained at 37 °C, 5% CO_2_ in F-12 DMEM containing 1 mM l-glutamine, 10% FBS, and 1% Pen/Strep as well as hygromycin B (300 µg/mL) and G418 (600 µg/mL) for CHO-K1 hCB1R cells or G418 (400 µg/mL) for CHO-K1 hCB2R cells^34,58^. For membrane preparation, cells were removed from flasks by scraping, centrifuged, and then frozen as a pellet at − 80 °C until required. Before use in a radioligand binding assay, cells were defrosted, diluted in Tris buffer (50 mM Tris–HCl and 50 mM Tris–base) and homogenized with a 1 mL hand-held homogenizer^34,58^. HitHunter (cAMP) and PathHunter (βarrestin2) CHO-K1 cells stably-expressing hCB1R from DiscoveRx (Eurofins, Fremont, CA) were maintained at 37 °C, 5% CO_2_ in F-12 DMEM containing 10% FBS and 1% penicillin–streptomycin with 800 µg/mL geneticin (HitHunter) or 800 µg/mL G418 and 300 µg/mL hygromycin B (PathHunter).

### CHO cell membrane preparation and radioligand displacement assay

CHO-K1 hCB1R and hCB2R cells were disrupted by cavitation in a pressure cell, and membranes were sedimented by ultracentrifugation, as described by Bolognini et al.^34,58^. The pellet was resuspended in TME buffer (50 mM Tris–HCl, 5 mM MgCl_2_, 1 mM EDTA, pH 7.4) and membrane proteins were quantified with a Bradford dye-binding method (Bio-Rad Laboratories, Mississauga, ON).

Assays were carried out with [^3^H]CP55,940 and Tris binding buffer (50 mM Tris–HCl, 50 mM Tris–base, 0.1% BSA, pH 7.4), total assay volume 2 mL, using the filtration procedure described previously by Baillie et al.^18^. The binding was initiated by the addition of transfected human CHO-K1 hCB1R and hCB2R cell membranes (50 µg protein per well). All assays were performed at 37 °C for 60 min before termination by the addition of ice-cold Tris binding buffer, followed by vacuum filtration using a 24-well sampling manifold (Brandel Cell Harvester; Brandel Inc, Gaithersburg, MD, USA) and Brandel GF/B filters that had been soaked in wash buffer at 4 °C for at least 24 h. Each reaction well was washed 6 times with 1.2 mL aliquots of Tris-binding buffer. The filters were air-dried overnight and then placed in 5 mL of scintillation fluid (Ultima Gold XR, PerkinElmer). Radioactivity was quantified by liquid scintillation spectrometry. Specific binding was defined as the difference between the binding that occurred in the presence and absence of 1 µM unlabelled CP55,940. The concentration of [^3^H]CP55940 used in our displacement assays was 0.7 nM.

### HitHunter cAMP assay

Inhibition of FSK-stimulated cAMP was determined using the DiscoveRx HitHunter assay in CHO-K1 hCB1R and hCB2R cells as we have described previously^15,23^. Briefly, cells (20,000 cells/well in low-volume 96 well plates) were incubated overnight in Opti-MEM containing 1% FBS at 37 °C and 5% CO_2_. Following this, Opti-MEM media was removed and replaced with cell assay buffer (DiscoveRx) and cells were co-treated at 37 °C with 10 µM FSK and ligands for 90 min. Following this, cAMP antibody solution and cAMP working detection solutions were added to cells according to the manufacturer’s directions (DiscoveRx), and cells were incubated for 60 min at room temperature^15,23^. cAMP solution A was added according to the manufacturer’s directions (DiscoveRx), and cells were incubated for an additional 60 min at room temperature before chemiluminescence was measured on a Cytation5 plate reader (top read, gain 200, integration time 10,000 ms).

### PathHunter βarrestin2 assay

βarrestin2 recruitment was determined using the DiscoveRx PathHunter assay in CHO-K1 hCB1R and hCB2R cells as we have described previously^15,23^. Briefly, cells (20,000 cells/well in low-volume 96 well plates) were incubated overnight in Opti-MEM containing 1% FBS at 37 °C and 5% CO_2_. Following this, cells were treated at 37 °C with ligands for 90 min. Following this, the detection solution was added to cells according to the manufacturer’s directions (DiscoveRx), and cells were incubated for 60 min at room temperature^15,23^. Chemiluminescence was measured on a Cytation5 plate reader (top read, gain 200, integration time 10,000 ms).

### Animals and tetrad testing

Adult male C57BL/6 mice aged 6–12 weeks (mean weight 22 ± 0.3 g) were purchased from Charles River Labs (Senneville, QC). Animals were group housed (3 per cage) with ad libitum access to food, water, and environmental enrichment and maintained on a 12 h light/dark cycle. Mice were randomly assigned to receive *i.p.* injections of vehicle (1:1:18 ethanol:emulphor:saline) or 0.1–100 mg/kg cannabinoid (*n* ≥ 6 per group)*.* All protocols were in accordance with the guidelines detailed by the Canadian Council on Animal Care^59,60^ and approved by the Animal Research Ethics Board and the Scientific Merit Review Committee for Animal Behaviour at the University of Saskatchewan. In keeping with the ARRIVE guidelines, power analyses were conducted to determine the minimum number of animals required for the study and animals were purchased—rather than bred—to limit animal waste, and all assessments of animal behaviour were made by individuals blinded to treatment group^60^. Catalepsy was assessed in the ring holding assay 10 min following injection. The mice were placed such that their forepaws clasped a 5 mm ring positioned 5 cm above the surface of the testing space. The length of time the ring was held was recorded (seconds). The trial was ended if the mouse turned its head or body, made 3 consecutive escape attempts, or at 60 s of immobility (i.e. MPE = 60 s). Internal body temperature was measured via rectal thermometer 12 min following injection. Anti-nociception was determined by assessing tail flick latency 15 min following injection. Mice were restrained with their tails placed ~ 1 cm into 52 °C water and the time until the tail was removed was recorded as tail flick latency (sec). Observations were ended at 20 s (i.e. MPE = 20 s). Locomotion was assessed in the OFT 1 h following injection. Mice were placed in an open space 90 cm × 90 cm, and the total distance was recorded for 5 min. Total distance travelled during 5 min (m) and time in the centre quadrant were measured with EthoVision XT (Noldus Information Technology Inc., Leesburg, VA).

### Statistical analyses

Data for [^3^H]CP55940 binding are shown as % change from maximal ^3^H bound (i.e. 100%). HitHunter cAMP, and PathHunter βarrestin2 data are shown as % of maximal CP55,940 response (i.e. 100%). Concentration–response curves (CRC) were fit using non-linear regression with variable slope (4 parameters) and used to calculate EC_50_, *E*_min_, and *E*_max_ (GraphPad, Prism, v. 8.0). CRCs were fit to the operational model of Black and Leff^25^ to calculate bias (∆∆LogR) according to previously described methods and using CP55,940 as the reference agonist^11^. In order to estimate bias for compounds with an EC_50_ > 10,000 nM, EC_50_ was set to 10,000 nM and *E*_max_ was set to the maximum observed response. In vivo data are presented as % MPE for catalepsy (MPE = 60 s) and anti-nociception (MPE = 20 s), °C for body temperature, and distance travelled (m) and % time in the centre quadrant in 5 min for OFT. Statistical analyses were conducted by one-way analysis of variance (ANOVA), as indicated in the figure legends, using GraphPad. Post-hoc analyses were performed using Tukey’s (one-way ANOVA) test. Homogeneity of variance was confirmed using Bartlett’s test. All data were evaluated for possible outliers using Grubb’s test in GraphPad. No outliers were removed. All results are reported as the mean ± the standard error of the mean (SEM) or 95% confidence interval (CI), as indicated. *p* values < 0.05 were considered to be significant.

## Supplementary information

Supplementary Figure S1.
