# Supplementary material for: In vitro and in vivo pharmacological activity of minor cannabinoids isolated from Cannabis sativa
Source: Sci Rep. 2020 Nov 23;10:20405. doi: 10.1038/s41598-020-77175-y (PMC7684313; doi:10.1038/s41598-020-77175-y)
Supplement: Supplementary file 1 — Supplementary Figure S1. [file 41598_2020_77175_MOESM1_ESM.docx]

**Supplementary Information for:** *In vitro* and *in vivo* pharmacological activity of minor cannabinoids isolated from *Cannabis sativa*

Ayat Zagzoog^1^, Kawthar A Mohamed^1^, Hye Ji (Jay) Kim^1^, Eunhyun D Kim^1^, Connor S Frank^1^, Tallan Black^1^, Pramodkumar D Jadhav^2^, Larry A Holbrook^3^, Robert B Laprairie^1,4*^

**Supplementary Figure 1.** Representative high performance liquid chromatography with diode-array detection (HPLC-DAD) chromatogram for ∆^9^-THCa obtained from Aurora Cannabis Inc. (Saskatoon, SK) demonstrating sample purity ≥98%. Because concern exists regarding the stability plant-derived cannabinoids, such as ∆^9^-THCa undergoing spontaneous decarboxylation, all compounds were aliquoted, stored at -80°C until use, and were used only once. Compounds were assessed for purity by HPLC-DAD using well-described methods [Wohlfarth et al. (2011); 57]. Image was constructed by authors using Empower 3 software for the Waters Chromatography Data System (v. 4.0)(Milford, MA).
